# The STATs in cell stress-type responses

**DOI:** 10.1186/1478-811X-2-8

**Published:** 2004-08-06

**Authors:** Andrew C Dudley, David Thomas, James Best, Alicia Jenkins

**Affiliations:** 1 The Department of Medicine, St. Vincent's Hospital, The University of Melbourne, Melbourne, Australia; 2 The Peter McCallum Cancer Center, Melbourne, Australia

## Abstract

In the early 1990's, a new cell signaling pathway was described. This new paradigm, now known as the JAK/STAT pathway, has been extensively investigated in immune-type cells in response to interferons and interleukins. However, recent evidence suggests that the JAK/STAT pathway also mediates diverse cellular responses to various forms of biological stress including hypoxia/reperfusion, endotoxin, ultraviolet light, and hyperosmolarity. The current literature describing the JAK/STAT pathway's role in cellular stress responses has been reviewed herein, but it is clear that our knowledge in this area is far from complete.

## Review

In multicellular organisms, every cell comprising every tissue, organ, or organ system constantly strives to maintain homeostasis in the face of destabilizing influences. Whether it is external stimuli such as toxic chemical exposure or changes in oxygen tension, or natural alterations in pH or osmolarity due to normal cellular metabolism, each cell in the body is equipped with the molecular machinery required to sense these environmental changes and respond to them. Because these stressful stimuli can impinge on normal cellular functioning, discrete cellular sensing mechanisms have evolved to maintain homeostasis. For example, ATP depletion caused by hypoxia activates AMPK which phosphorylates multiple downstream targets to switch the cell from a mainly anabolic to catabolic state [1]. Similarly, activation of SAPK/JNK by stressors such as ultraviolet (UV) light results in activation of well-defined molecular targets [reviewed in [2]].

One interesting question is how do cells translate diverse stressful stimuli into activation of specific molecular pathways? Cellular signaling from membrane to nucleus is typically accomplished through ligand/receptor activation of intracellular second messengers. In many cases these second messengers are kinases which phosphorylate substrates leading to a cascade by which successive macromolecules are triggered. Ultimately, a terminal transcription factor is activated which then translocates to the nucleus to activate specific target genes. This type of cellular signaling has been well-characterized following receptor activation by polypeptide ligands, but some forms of cellular stress such as hypoxia cause activation of specific molecular pathways in the apparent absence of ligand to receptor stimulation. Therefore, there must be alternative routes for direct activation of target genes that circumvents the canonical ligand/receptor/second messenger cascade. One such pathway that may transmit signals to the nucleus by this alternative route is the Janus Activated Kinase/Signal Transducer and Activator of Transcription family of transcription factors (JAK/STAT).

The JAK/STAT pathway includes seven functionally related, latent transcription factors (STAT) and four non-receptor tyrosine kinases (JAK) [reviewed in [3]]. In the typical JAK/STAT paradigm, a cytokine binding to its receptor results in activation of receptor-associated JAKs. JAKs then phosphorylate the cytoplasmic receptor chains creating docking sites for recruited STATs. Finally, STATs are phosphorylated on tyrosine by JAKs, dimerize, and then translocate to the nucleus to activate specific target genes. Because the second messenger (STAT) is also the terminal transcription factor, in many ways the JAK/STAT pathway represents a streamlined apparatus for cellular signaling. Thus, the JAK/STAT pathway differs from many signaling cascades in that the usual system of multiple sequential signaling molecules is bypassed.

While signaling through the canonical JAK/STAT pathway has been well-characterized in immune-type cells in response to interleukins and interferons, there is emerging evidence that STATs also mediate cellular responses to various forms of cellular stress. These findings, in addition to mounting evidence suggesting that some STATs are phosphorylated on serine by members of the MAPK family, imply that alternative mechanisms for STAT activation exist. Further, these alternative means of activation may lead to different outcomes with regards to STAT signaling. For example, STAT3 was recently shown to be serine phosphorylated by JNK during UV stress which had a predominately inhibitory role on STAT3 transcriptional activity [4]. Could these alternative routes of STAT activation account for STAT's "yin and yang" type properties whereby depending on the type of cell stressor, either cell death or cell survival pathways are activated? Although interferons themselves can regulate cellular responses to exogenous stressors such as infection through the canonical JAK/STAT pathway, this literature review will focus mainly on those alternative mechanisms of JAK/STAT signaling during cellular stress.

## STATs and cellular stress

Some of the earliest studies implicating STATs in mediating cell stress responses were performed in cells exposed to UV light. In mouse embryonic fibroblasts (MEFs), UV light treatment resulted in phosphorylation of serine 727 in STAT1 via p38 MAPK [5,6]. Further analysis revealed that STAT1 could be phosphorylated directly by p38 MAPK *in vitro*. Thus, the MAPK and STAT pathways appear to converge during periods of cellular stress. In another study, UV light caused STAT1 tyrosine phosphorylation, nuclear accumulation, and DNA binding in keratinocytes [7]. Together, these studies raise the possibility that STATs can be activated in a ligand-independent manner during cellular stress, resulting in the activation of STAT-dependent target genes.

Cellular stress can also occur in disease states such as diabetes, which is characterized by vascular dysfunction. For example, prolonged elevated glucose can act as a cell stressor through multiple pathways including hyperosmolarity [8], protein kinase C (PKC) activation [9], and oxidative damage [10]. Recent studies have determined that constitutive JAK/STAT phosphorylation was elevated in cultured smooth muscle-like mesangial cells treated with high glucose [11]. Furthermore, in these same cells, angiotensin stimulation of STATs was prolonged by high glucose treatment [12,13]. The authors of these studies related these findings to TGFβ-induced extracellular matrix accumulation because collagen and fibronectin secretion could be inhibited with STAT anti-sense RNAs. This implies that the JAK/STAT pathway may play a role in basement membrane thickening observed in diabetic nephropathy and retinopathy. These studies were some of the first to suggest that perturbed JAK/STAT signaling could play a role in disease states like diabetes and possibly link glucose-mediated cell stress and the JAK/STAT pathway with diabetic sequelae.

Hyperosmotic stress can also activate STATs. For example, in the slime mold *Dictyostelium*, hyperosmotic stress leads to STAT1 phosphorylation without any known involvement of JAK or MAPK [14]. But mammalian cells also utilize STATs during hyperosmotic stress. In one report, sorbitol-induced hyperosmolarity was shown to cause JAK1, JAK2, and TYK2 phosphorylation and subsequent activation of STAT1 and STAT3 in various cell types; this led to the formation of STAT1/STAT3 complexes with the m67SIE oligonucleotide from the c-fos promoter [15]. Interestingly, these authors speculate that the hyperosmotic signal occurred independently of gp130. This suggests an alternative pathway by which JAKs may be activated beyond the canonical JAK/STAT route. In agreement with this study, hyperosmotic shock in COS-7 cells was shown to lead to tyrosine phosphorylation of STAT1 in a MKK6/p38-dependent pathway [16]. In this case, STAT1 but not JAK1 phosphorylation could be inhibited by genistein (a non-specific tyrosine kinase inhibitor) leading the authors to conclude that a tyrosine kinase distinct from JAK1 (possibly novel) represented the link between hypertonicity and STAT activation.

The most well-investigated reports linking cellular stress with the JAK/STAT pathway are studies in cardiomyocytes undergoing hypoxia/reperfusion. Like UV light, hypoxia/reperfusion led to p38 MAPK phosphorylation followed by serine 727 phosphorylation of STAT1 which was associated with activation of pro-apoptotic FAS/FASL and caspase-1 [17]. It was concluded that Fas and caspase-1 expression were directly STAT-1 dependent because their expression could be inhibited by STAT1 anti-sense RNAs. Thus, STAT1-dependent FAS activation plays a leading role in cardiomyocyte death during hypoxia/reperfusion injury [18] and as the authors point out, inhibition of this pathway may prove to be cardioprotective following ischemic insult [19]. Interestingly, while these studies showed that both tyrosine 701 and serine 727 of STAT1 were phosphorylated in response to hypoxia/reperfusion, only phosphorylation on serine was required for FAS expression. Because serine phosphorylation alone is not sufficient for direct DNA binding, these results indicate alternative pathways by which STAT1 may activate target genes during stress. For example, serine phosphorylated STAT1 may associate with other scaffolding proteins as it does in the case of MCM5 [20], BRCA1 [21], or HSF [22] and act instead as a transcriptional co-activator, rather than a direct activator of target genes [23].

*In vivo *models of hypoxia/reperfusion also implicate STAT5 as a player in responses to cellular stress. But unlike STAT1, STAT5 is thought to be mainly protective by activating anti-apoptotic signals. For example, Yamaura *et al*. report that genetic deletion of STAT6 but not the STAT5A causes resistance to myocardial ischemia/reperfusion injury [24]. This resistance was thought to be related to two distinct STAT5A-mediated pathways: one involving a Src/STAT5/PI-3 kinase/Akt pathway, and the other a direct JAK2/STAT5A pathway. Like STAT5, STAT3 was also shown to be protective against cardiac ischemia/reperfusion injury through a JAK2/STAT3-dependent mechanism involving up-regulation of anti-apoptotic Bcl2 and down-regulation of pro-apoptotic Bax [25,26]. Studies in our laboratory indicate that in microvascular endothelial cells, hypoxia caused an increased tyrosine phosphorylation of JAK2, down-regulation of FAS/FASL, and that AG490 (a JAK2 inhibitor) de-repressed FAS transcription (unpublished observations). This may suggest a possible mechanism whereby activated JAK2 could mediate protection during ischemia in endothelial cells by repressing pro-apoptotic FAS transcription through downstream STAT3 or STAT5. Intriguingly, a complex of STAT3 and c-Jun was recently shown to be a FAS repressor [27,28] but it remains to be determined if STAT5 or other STATs can behave like STAT3 and act as transcriptional repressors of pro-apoptotic genes during cellular stress. STAT5 nuclear translocation and DNA binding to the GAS (γ-activated site) implicates STAT5 in hypoxic stress responses, but the biological significance of this observation is not yet clear [29].

Although not as well studied as hypoxia/reperfusion or osmotic stress, reactive oxygen species have also been shown to activate the JAK/STAT pathway. Oxidative stress, such as might occur in diabetes and cardiovascular disease, was shown to activate HSP70 in smooth muscle cells in a JAK-dependent manner [30]. This response is thought to aid in adapting these cells to oxidative damage. Recently it was shown that STAT1 forms a complex with HSF-1 to activate the HSP promoter while STAT3 filled just the opposite role [22,31]. Thus, it appears that STAT1 and STAT3 can perform entirely different functions with regard to cellular stress-type responses. Other studies have determined that peroxide treatment resulted in STAT3 tyrosine phosphorylation and nuclear translocation [32] and JAK2, STAT1, and STAT3 were activated by oxidized LDL [33].

Taken together, the studies reviewed herein support a role for the JAK/STAT pathway in various forms of cellular stress and relate perturbed JAK/STAT signaling to potential disease states. However, consistent with some of the current ideas about STAT biology, it is clear that cellular stress seems to activate STATs in ways that can be both detrimental to and supportive of cell survival. For example, STAT1 activation by hypoxia-reperfusion injury activates cell death pathways, while STAT5 activation by the same type of stressor seems to promote cell survival pathways. Thus, STATs may have evolved to fulfil both sides of a "yin and yang" type mechanism where either death or survival pathways can be activated depending on the strength or type of cellular stressor [34]. This may be especially true in endothelial cells, which seem to be able to resist short-term hypoxic-stress compared to other cell types but die by apoptosis following prolonged exposure to hypoxia [35].

The most intriguing aspect of many cellular stress-activated pathways is the apparent absence of ligand-to-receptor stimulation. But the cell must somehow "sense" changes in the external milieu and transmit these signals to the nucleus. How does it do this? Two such examples of this type of cellular sensing are activation of a well-described transcription factor known as HIF-1α (hypoxia-inducible factor), and another is the cellular thermostat called HSF (heat shock factor). In the case of HIF, enzymatic modification by an enzyme requiring oxygen as a cofactor is responsible for HIF activation and switching on of target genes when cells are stressed by low oxygen [36]. For HSF multiple stressors such as ATP depletion, ischemia, and intracellular acidosis lead to HSF phosphorylation, unfolding, and its translocation to the nucleus to activate target genes [37]. So these powerful signal transducing pathways are somehow activated directly presumably without ligand stimulation. This is probably the most speedy and efficient way of activating downstream target genes to promote cell survival.

## Conclusions

**Figure 1:**
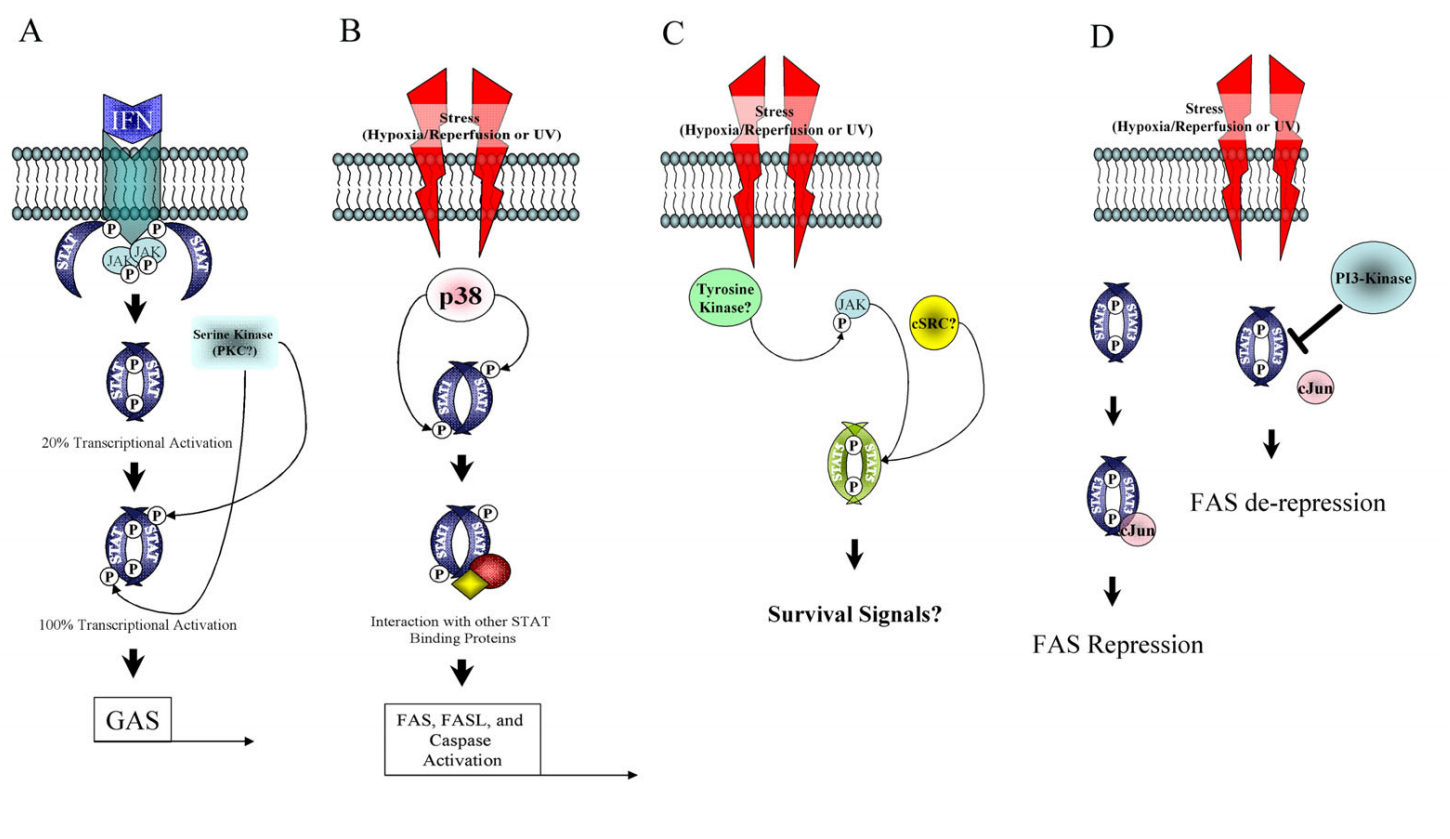
**Role of STATs in cell stress responses. **(A) Autocrine IFN may activate JAK/STAT through the canonical pathway. This activation would involve tyrosine phosphorylation of STAT by JAK resulting in STAT dimers which are 20% transcriptionally active. This process is thought to "prime" STATs for serine phosphorylation by an IFN-inducible serine kinase (possibly PKC) [42]. Both tyrosine and serine phosphorylation results in a 100% transcriptionally active STAT1 dimer. (B) Hypoxia-reperfusion injury may directly activate p38 MAPK which phosphorylates STAT1 on SER727. Serine phosphorylated STAT could then participate in protein-protein interactions with other STAT binding proteins and activate the expression of pro-apoptotic genes like FAS. (C) In this case, hypoxia-reperfusion may activate STAT5 resulting in activation of cell survival pathways. STAT5 activation by hypoxia may be mediated by JAK2 and a STAT5/cSrc/PI-3 kinase/Akt pathway. (D) STAT3 may act as a constitutive FAS repressor, but FAS is de-repressed during UV stress which may involve STAT3 inhibition by PI3-kinase/Akt.

Is it possible that STATs can also act as a cellular rheostat of various stressors? Recent suggestions that STATs can be post-translationally modified in ways other than phosphorylation (e.g. acetylation, methylation, and ubiquitination) make this a possibility [38]. For example, changes in the intracellular redox environment by low oxygen tension may modify STAT conformation leading to enhanced availability of its active centers [15]. This change may facilitate interactions with other STAT-modifying proteins such as p38 MAPK. Alternatively, other previously unknown STAT pathways may be activated during cellular stress, altering its transcriptional capacity. These might include STAT association with other second messengers such as PI-3 kinase and Akt but also STAT upstream activation by molecules like Src [24]. Figure 1 summarizes the potential role of STATs in cellular stress.

Finally, while our understanding of a stress-related p38/STAT1 (pSER) pathway seems to be taking shape, very few studies have investigated the role of serine phosphorylation of other STATs and what the upstream kinase (s) may be during cellular stress. Future studies might focus on identifying whether serine phosphorylation is common to other STATs during cellular stress and how this might relate to activation or inactivation of target genes. Other questions to be answered are what is the function of unphosphorylated STAT dimers found in the nucleus of unstimulated cells and how might other STAT post-translational modifications (other than phosphorylation) mediate STAT signaling beyond the canonical JAK/STAT pathways [39-41]. Answers to these questions may help to begin to unravel the complex nature of STAT signaling and how some of the alternative routes of STAT activation are related to cellular stress-activated pathways. Ultimately, modulation of the JAK/STAT pathway *in vivo *may prove to be of therapeutic value.
